# Institutional deliveries and perinatal and neonatal mortality in Southern and Central India

**DOI:** 10.1186/1742-4755-12-S2-S13

**Published:** 2015-06-08

**Authors:** Shivaprasad S Goudar, Norman Goco, Manjunath S Somannavar, Sunil S Vernekar, Ashalata A Mallapur, Janet L Moore, Dennis D Wallace, Nancy L Sloan, Archana Patel, Patricia L Hibberd, Marion Koso-Thomas, Elizabeth M McClure, Robert L Goldenberg

**Affiliations:** 1 Women's and Children's Health Research Unit, KLE University's Jawaharlal Nehru Medical College, Belgaum, Karnataka, India; 2 RTI International, Durham, NC, USA; 3 S Nijalingappa Medical College, Bagalkot, Karnataka, India; 4 Christiana Care Health Services, Newark, DE, USA; 5 Latta Medical Research Foundation, Nagpur, India; 6 Massachusetts General Hospital, Boston, MA, USA; 7 Eunice Kennedy Shriver National Institute of Child Health and Human Development, Bethesda, MD, USA; 8 Department of Obstetrics and Gynecology, Columbia University School of Medicine, New York, NY, USA

**Keywords:** Perinatal mortality, stillbirth, neonatal mortality, institutional delivery, household surveillance, India

## Abstract

**Background:**

Skilled birth attendance and institutional delivery have been advocated for reducing maternal, perinatal and neonatal mortality (PMR and NMR). India has successfully implemented various strategies to promote skilled attendance and incentivize institutional deliveries in the last 5 years.

**Objectives:**

The study evaluates the trends in institutional delivery, PMR, NMR, and their risk factors in two *Eunice Kennedy Shriver* NICHD Global Network for Women’s and Children’s Health Research sites, in Belgaum and Nagpur, India, between January 2010 and December 2013.

**Design/methods:**

Descriptive data stratified by level of delivery care and key risk factors were analyzed for 36 geographic clusters providing 48 months of data from a prospective, population-based surveillance system that registers all pregnant permanent residents in the study area, and their pregnancy outcomes irrespective of where they deliver. Log binomial models with generalized estimating equations to control for correlation of clustered observations were used to test the trends significance

**Results:**

64,803 deliveries were recorded in Belgaum and 39,081 in Nagpur. Institutional deliveries increased from 92.6% to 96.1% in Belgaum and from 89.5% to 98.6% in Nagpur (both p<0.0001); hospital rates increased from 63.4% to 71.0% (p=0.002) and from 63.1% to 72.0% (p<0.0001), respectively. PMR declined from 41.3 to 34.6 (p=0.008) deaths per 1,000 births in Belgaum and from 47.4 to 40.8 (p=0.09) in Nagpur. Stillbirths also declined, from 22.5 to 16.3 per 1,000 births in Belgaum and from 29.3 to 21.1 in Nagpur (both p=0.002). NMR remained unchanged.

**Conclusions:**

Significant increases in institutional deliveries, particularly in hospitals, were accompanied by reductions in stillbirths and PMR, but not by NMR.

## Background

Of the 7.6 million annual deaths worldwide in children under 5 years of age, 3.1 million occur in the first 28 days of life [1]. India accounts for more of these neonatal deaths and the 2.6 million annual stillbirths than any other country [1,2]. While remarkable progress has been made in reducing mortality in children under five years old, many countries have not met the Millennium Development Goal (MDG) 4 target to reduce child mortality by two-thirds by 2015 because similar success has not been achieved in reducing peri- and neonatal mortality (PMR and NMR) [1,3].

Most maternal, perinatal and neonatal complications and mortality occur at or shortly after labor and delivery [4-6]. Skilled birth attendance and an institutional environment capable of providing effective obstetric and neonatal care are needed to significantly reduce maternal deaths, stillbirths and early neonatal deaths. In support of the United Nations MDGs to reduce child mortality and improve maternal health worldwide [7], increasing skilled birth attendance and births in hospitals and health centers have been recommended [8,9]. Some countries, such as India, where home birth was common until recently, have taken successful steps aimed to implement this strategy through programs such as *Janani Suraksha Yojana* (*JSY*), a conditional cash transfer scheme the Government of India uses to incentivize deliveries in government health facilities [10]. Simultaneously, efforts are underway to improve the delivery and newborn care practices of birth attendants working at these health facilities [11,12]. However, to date, few studies have assessed the potential impact of these activities on maternal, perinatal and maternal mortality.[10,13-15]

In a prospective study, we sought to evaluate the trends in institutional delivery and perinatal and neonatal mortality rates in two regions, Belgaum and Nagpur, over a four-year period from January 2010 to December 2013. Belgaum is located in Karnataka State, in Southern India. Nagpur is located in Maharashtra State in Central India. While Belgaum and Nagpur districts have similar population sizes, 4.8 and 4.6 million, [16,17] respectively, the two sites have distinct population characteristics. Over one-third of reproductive age women in Karnataka have no formal education, and 54% of reproductive age women and 10% of men had no employment in the 12 months prior to the most recent National Family Health Survey [18]. In comparison, 24% of reproductive age women in Maharashtra have no formal education, and 52% of reproductive age women and 13% of men had no employment in the 12 months prior to the most recent National Family Health Survey [18]. The adult literacy rate in Belgaum is 73% compared with 88% in Nagpur, and the male: female population ratio is 1.03 in Belgaum and 1.05 in Nagpur [16,17]. The sample age and parity were also distinct, with more young but fewer nulliparous women in Belgaum than Nagpur (Table 1).

**Table 1:** Maternal demographic and clinical characteristics

	Belgaum				Nagpur			
	
	2010	2011	2012	2013	2010	2011	2012	2013
Deliveries, N	16,345	16,909	17,356	14,193	10,097	9,572	9,521	9,891

Maternal age, N (%)	16,321	16,909	17,355	14,193	10,089	9,571	9,512	9,881

< 20	1,731 (10.6)	1,686 (10.0)	1,719 (9.9)	1,334 (9.4)	211 (2.1)	178 (1.9)	192 (2.0)	189 (1.9)

20-35	14,557 (89.2)	15,186 (89.8)	15,609 (89.9)	12,836 (90.4)	9,861 (97.7)	9,368 (97.9)	9,286 (97.6)	9,660 (97.8)

> 35	33 (0.2)	37 (0.2)	27 (0.2)	23 (0.2)	17 (0.2)	25 (0.3)	34 (0.4)	32 (0.3)

Maternal education, N (%)	16,160	16,755	17,283	14,181	10,093	9,571	9,497	9,872

No formal education	3,666 (22.7)	3,398 (20.3)	2,981 (17.2)	2,085 (14.7)	391 (3.9)	288 (3.0)	268 (2.8)	250 (2.5)

Primary	5,751 (35.6)	5,556 (33.2)	5,474 (31.7)	4,394 (31.0)	1,845 (18.3)	1,633 (17.1)	1,613 (17.0)	1,692 (17.1)

Secondary	5,560 (34.4)	6,275 (37.5)	7,009 (40.6)	5,986 (42.2)	6,009 (59.5)	5,793 (60.5)	5,686 (59.9)	5,753 (58.3)

University +	1,183 (7.3)	1,526 (9.1)	1,819 (10.5)	1,716 (12.1)	1,848 (18.3)	1,857 (19.4)	1,930 (20.3)	2,177 (22.1)

Parity, N (%)	16,318	16,906	16,918	14,192	10,097	9,571	9,517	9,888

0	6,738 (41.3)	7,257 (42.9)	7,473 (44.2)	6,153 (43.4)	4,897 (48.5)	4,707 (49.2)	4,612 (48.5)	4,598 (46.5)

1-2	8,407 (51.5)	8,496 (50.3)	8,392 (49.6)	7,214 (50.8)	4,902 (48.5)	4,621 (48.3)	4,667 (49.0)	5,047 (51.0)

> 2	1,173 (7.2)	1,153 (6.8)	1,053 (6.2)	825 (5.8)	298 (3.0)	243 (2.5)	238 (2.5)	243 (2.5)

At least one ANC visit, N (%)	16,341	16,901	17,215	14,148	10,096	9,556	9,518	9,883

Yes	16,308 (99.8)	16,886 (99.9)	17,205 (99.9)	14,142 (100.0)	10,095 (100.0)	9,556 (100.0)	9,516 (100.0)	9,869 (99.9)

No	33 (0.2)	15 (0.1)	10 (0.1)	6 (0.0)	1 (0.0)	0 (0.0)	2 (0.0)	14 (0.1)

## Methods

This study was conducted in Belgaum, Karnataka State and Nagpur, Maharashtra State, India in the *Eunice Kennedy Shriver* Global Network for Women’s and Children’s Health Research study clusters as part of the Maternal Newborn Health Registry (MNHR) study [13]. The MNHR is a prospective, population-based surveillance system of pregnant women and their pregnancy outcomes. Study clusters are distinct geographical areas, which were defined initially with approximately 300-500 births per year. All consenting pregnant women who are permanent residents of the clusters are prospectively enrolled into the registry irrespective of their delivery location (99.9% of eligible women in both populations consent to participate). Study visits are completed at delivery and 42 days postpartum to record outcomes by Registry Administrators, who are medical officers or health workers working at the Primary Health Centers for the Ministry of Health. The MNHR 2010-2013 data exclude deliveries in women lost to follow up prior to delivery, or those with deliveries resulting in miscarriages/medically terminated pregnancies and those weighing <1000g at birth.

The Belgaum and Nagpur sites also benefit from an annual household survey to enroll all married women of reproductive age (i.e. 15-49 years) residing within the clusters, which is an adaptation of the Ministry of Health (MOH) survey but administered independently under the supervision of the site investigators. This household surveillance enables identification of women who are currently pregnant, have undergone sterilization, as well as those who are likely to conceive in the ensuing year. It is also used to estimate the crude birth rate and projected enrollment for the forthcoming year. Completeness of coverage of the registry enrollment from the catchment area is assessed by comparing the projected and actual enrollments. The surveillance system has been previously described in detail [19].

Institutional birth was defined as delivery at either a hospital or clinic (primary health center or community health center). Community births included those delivered at home (generally the mother’s home or birth attendant’s home). Stillbirth rates were defined as deaths prior to delivery among all births ≥ 20 weeks gestation. We also examined neonatal death rates, including early (< 7 day) neonatal deaths and 28-day (deaths <28 days), per 1000 live births. The present analysis includes 16 of the 24 study clusters in Belgaum that contributed data to all 48 months included in the analysis and 20 clusters in Nagpur (all of which contributed 48 months of data). Descriptive statistics were performed. Analyses were stratified by level of delivery care and key risk factors. We modeled mortality risk and the prevalence of delivery location and calculated point and interval estimates of risk ratios using multivariable generalized linear regression models with a binomial distributional assumption and a log link; we used generalized estimating equations to account for correlation of outcomes within clusters to assure appropriately sized p-values and confidence intervals. To evaluate changes over time, we modelled year of delivery as a categorical value and tested for changes between Year 2010 and Year 2013 with a simple difference contrast and tested for trends across time with an orthogonal polynomial linear contrast. All analyses were conducted using SAS v.9.3 (Cary, NC).

## Results

Between January 2010 and December 2013, 64,803 deliveries were recorded in the 16 clusters providing 48 months of data in MNH Registry in Belgaum and 39,081 in the 20 Nagpur clusters (Table 1). Of these deliveries, 99.8% had follow-up visits available. During this time period, fewer women with no formal education gave birth in Belgaum (22.7% to 14.7%) with a similar trend in Nagpur, dropping from 3.9% to 2.5%. There was a steady decrease in births to women of parity greater than 2 in both sites (Belgaum: 7.2% to 5.8% and Nagpur: 3.0% to 2.5%). Maternal age at birth increased in Belgaum, with births among women below the age of 20 years steadily decreasing from 10.6% to 9.4%, whereas there was no change in maternal age at birth in Nagpur. The proportion of deliveries with at least 1 antenatal care visit was consistently greater than 99% each year for deliveries registered in both sites.

**Figure 1:**
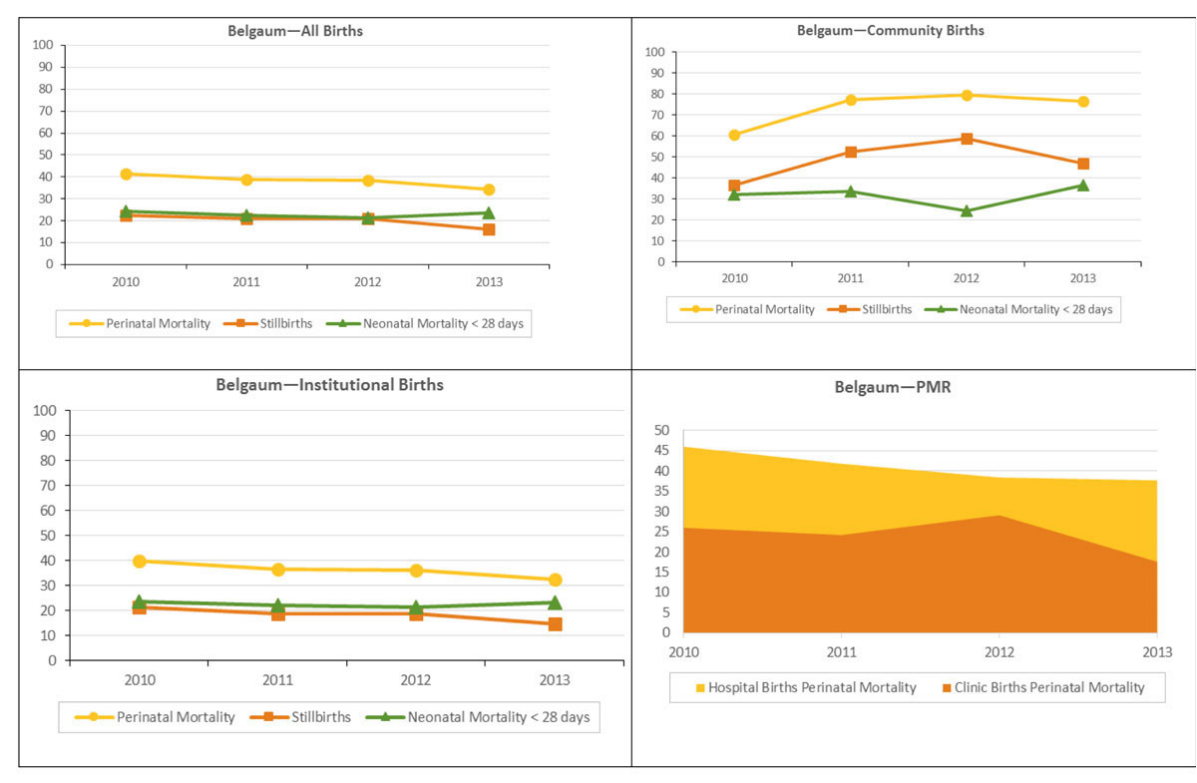
PMR, stillbirths and NMR <28 days: Belgaum

Institutional delivery (hospital and clinic) increased from 92.1% to 96.0% in Belgaum and from 89.7% to 98.7% in Nagpur. The increase in hospital deliveries (63.6% to 71.6% in Belgaum, and 64.1% to 73.0% in Nagpur) accounted for most of this shift, with clinic deliveries remaining stable or declining (Table 2, Figure 1). Home births declined by about 50% in Belgaum (7.9% to 4.0%) and by nearly 90% in Nagpur (10.3% to 1.3%). Consistent with the shift in birth location, in 2013 more births were attended by physicians or nurses/nurse midwives than in 2010 in both sites (Belgaum: 2010: 92.8%; 2013: 96.6%; Nagpur: 2010: 91.0%; 2013: 98.9%). Physician attended deliveries increased (Belgaum: 2010: 59.2%; 2013: 61.0%; Nagpur: 2010: 56.1%; 2013: 64.3%). Deliveries attended by a traditional birth attendant decreased, from 3.6% in Belgaum and 7.4% in Nagpur in 2010 to less than 1% in both locations in 2013, while the percent attended by nurse midwives was fairly stable. Home deliveries with no health provider or a family member fell from 3.6% to 2.6% in Belgaum and declined from 1.6% to 0.6% in Nagpur. The increase in institutional deliveries and skilled birth attendance was accompanied by an increase in cesarean sections in Belgaum (2010: 9.9% to 2013: 18.6%) and in Nagpur (2010: 17.6% to 2013: 23.0%).

**Table 2:** Delivery characteristics

	Belgaum				Nagpur			
	
	2010	2011	2012	2013	2010	2011	2012	2013
Deliveries, N	16,345	16,909	17,356	14,193	10,097	9,572	9,521	9,891

Delivery location, N (%)	16,317	16,902	17,350	14,189	10,092	9,565	9,514	9,885

Institutional	15,032 (92.1)	15,935 (94.3)	16,524 (95.2)	13,616 (96.0)	9,048 (89.7)	9,213 (96.3)	9,324 (98.0)	9,757 (98.7)

Hospital	10,381 (63.6)	11,316 (67.0)	12,851 (74.1)	10,154 (71.6)	6,464 (64.1)	6,483 (67.8)	6,388 (67.1)	7,215 (73.0)

Clinic	4,651 (28.5)	4,619 (27.3)	3,673 (21.2)	3,462 (24.4)	2,584 (25.6)	2,730 (28.5)	2,936 (30.9)	2,542 (25.7)

Community (Home or other)	1,285 (7.9)	967 (5.7)	826 (4.8)	573 (4.0)	1,044 (10.3)	352 (3.7)	190 (2.0)	128 (1.3)

Birth attendant, N (%)	16,342	16,903	17,350	14,189	10,092	9,565	9,516	9,885

Physician	9,668 (59.2)	9,602 (56.8)	10,712 (61.7)	8,649 (61.0)	5,659 (56.1)	5,521 (57.7)	5,849 (61.5)	6,356 (64.3)

Nurse/Midwife/HW	5,496 (33.6)	6,420 (38.0)	5,895 (34.0)	5,046 (35.6)	3,525 (34.9)	3,731 (39.0)	3,486 (36.6)	3,416 (34.6)

TBA	586 (3.6)	352 (2.1)	227 (1.3)	126 (0.9)	750 (7.4)	206 (2.2)	95 (1.0)	52 (0.5)

Family/Other	592 (3.6)	529 (3.1)	516 (3.0)	368 (2.6)	158 (1.6)	107 (1.1)	86 (0.9)	61 (0.6)

Delivery mode, N (%)	16,342	16,903	17,350	14,189	10,052	9,538	9,493	9,864

Vaginal	14,658 (89.7)	14,707 (87.0)	14,647 (84.4)	11,466 (80.8)	8,184 (81.4)	7,753 (81.3)	7,517 (79.2)	7,361 (74.6)

Vaginal assisted	61 (0.4)	41 (0.2)	54 (0.3)	80 (0.6)	103 (1.0)	28 (0.3)	66 (0.7)	236 (2.4)

C-section	1,623 (9.9)	2,155 (12.7)	2,649 (15.3)	2,643 (18.6)	1,765 (17.6)	1,757 (18.4)	1,910 (20.1)	2,267 (23.0)

Bag and mask resuscitation, N (%)	16,063	16,824	17,271	14,118	10,092	9,501	9,395	9,913

Yes	785 (4.9)	842 (5.0)	741 (4.3)	447 (3.2)	241 (2.4)	359 (3.8)	364 (3.9)	400 (4.0)

No	15,278 (95.1)	15,982 (95.0)	16,530 (95.7)	13,671 (96.8)	9,851 (97.6)	9,142 (96.2)	9,031 (96.1)	9,513 (96.0)

Multiple birth, N (%)	16,337	16,903	17,350	14,189	10,092	9,564	9,500	9,861

Yes	100 (0.6)	115 (0.7)	137 (0.8)	104 (0.7)	84 (0.8)	61 (0.6)	71 (0.7)	83 (0.8)

No	16,237 (99.4)	16,788 (99.3)	17,213 (99.2)	14,085 (99.3)	10,008 (99.2)	9,503 (99.4)	9,429 (99.3)	9,778 (99.2)

The percent of multiple gestation births remained <1%. The sex ratio (male/female) for both sites was relatively stable during this period with Nagpur experiencing a slight decrease from 1.14 to 1.06 (Table 3).

**Table 3:** Infant characteristics

	Belgaum				Nagpur			
	
	2010	2011	2012	2013	2010	2011	2012	2013
Births, N	16,445	17,024	17,497	14,295	10,178	9,633	9,590	9,975

Gender, N (%)	16,431	17,006	17,483	14,287	10,113	9,572	9,539	9,925

Male	8,434 (51.3)	8,896 (52.3)	9,101 (52.1)	7,361 (51.5)	5,380 (53.2)	4,964 (51.9)	5,002 (52.4)	5,109 (51.5)

Female	7,997 (48.7)	8,110 (47.7)	8,382 (47.9)	6,926 (48.5)	4,733 (46.8)	4,608 (48.1)	4,537 (47.6)	4,816 (48.5)

Sex ratio (M/F)	1.05	1.10	1.09	1.06	1.14	1.08	1.10	1.06

**Figure 2:**
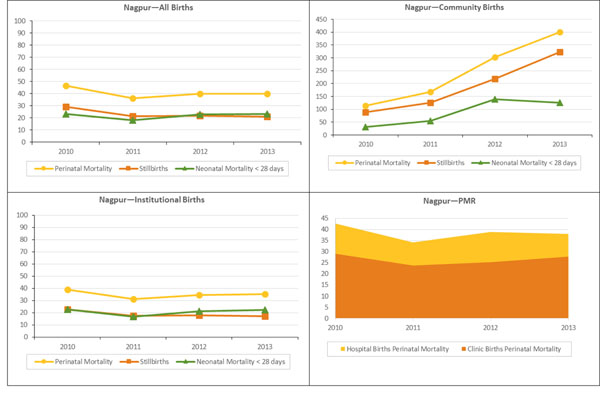
PMR, stillbirths and NMR <28 days: Nagpur

The perinatal mortality, stillbirth, and neonatal mortality rates overall and stratified by location are illustrated in figures 1 and 2. These rates were substantially higher in the community than in institutional deliveries. Community PMR increased from 60.7 to 76.7 per 1000 births in Belgaum and from 114.1 to 400.0 deaths per 1000 births in Nagpur; however the number of community births was small in Nagpur. Among institutional deliveries, PMR declined in Belgaum from 39.9 to 32.6 deaths per 1000 births; the decline was observed in both hospital and clinic deliveries. The PMR fluctuated between 38.9 (2010) and 35.3 (2013), in hospitals and in clinics, in Nagpur. Neonatal mortality (through 28 days of life) remained stable in institutional births in Belgaum and Nagpur but increased in community births in both sites, and in clinic births in Nagpur.

Adjusted risk estimates for trends in deliveries and mortality stratified by location are presented in Table 4. There was a steady decline in the perinatal mortality rate in Belgaum, from 41.3 to 34.6 deaths per 1,000 births (p=0.008) and in Nagpur from 47.4 to 40.8 deaths per 1,000 births (p=0.09) between 2010 and 2013 (Table 4). The stillbirth rate also declined for both sites, from 22.5 to 16.3 per 1000 births in Belgaum and from 29.3 to 21.1 per 1000 births in Nagpur (both p=0.002). In Belgaum, the early neonatal mortality rate (within 7 days of delivery) declined marginally over this period from 19.3 deaths per 1000 live births to 18.6 deaths per 1000 live births (p=0.62). In contrast, at the Nagpur site, the rate of early neonatal mortality rose from 18.7 deaths per 1000 live births in 2010 to 20.4 deaths per 1000 live births in 2013 (p=0.03). The 28-day neonatal mortality rate remained relatively stable in both Belgaum (2010: 24.2; 2011: 22.6; 2012: 21.4; 2013: 23.8 per 1000 live births) and in Nagpur (2010: 24.5; 2011:19.0; 2012: 24.0; 2013: 24.4).

**Table 4:** Trends in Deliveries and Mortality Rates by Location

	Adjusted Risk Estimate (95% CI)				
		
	2010	2011	2012	2013	P-value for 2010 - 2013 trend test
Belgaum					

Institutional Deliveries, n (%)	92.6 (90.2, 95.0)	94.6 (92.8, 96.4)	95.5 (94.0, 97.0)	96.1 (94.8, 97.4)	<.0001

Hospital Deliveries, n (%)	63.4 (54.7, 73.5)	66.3 (58.6, 75.1)	73.6 (66.5, 81.4)	71.0 (63.8, 78.9)	0.0020

Perinatal mortality, rate/1000 births	41.3 (36.1, 47.3)	39.0 (33.4, 45.5)	38.5 (34.8, 42.7)	34.6 (31.8, 37.6)	0.0078

Stillbirths, rate/1000 births	22.5 (19.0, 26.7)	20.9 (17.4, 25.1)	20.8 (18.6, 23.3)	16.3 (14.2, 18.7)	0.0017

NMR < 7 days, rate/1000 live births	19.3 (16.6, 22.4)	18.5 (15.5, 22.1)	18.1 (15.7, 20.8)	18.6 (16.7, 20.6)	0.6226

Neonatal mortality < 28 days, rate/1000 live births	24.2 (21.0, 27.8)	22.6 (18.3, 27.8)	21.4 (18.5, 24.8)	23.8 (21.5, 26.3)	0.6496

Nagpur					

Institutional Deliveries, n (%)	89.5 (86.0, 93.2)	96.3 (95.5, 97.1)	97.9 (97.4, 98.5)	98.6 (98.3, 98.9)	<.0001

Hospital Deliveries, n (%)	63.1 (57.4, 69.4)	66.9 (61.8, 72.5)	66.1 (60.5, 72.2)	72.0 (66.6, 77.8)	<.0001

Community Deliveries, n (%)					

Perinatal mortality, (rate/1000 births)	47.4 (41.9, 53.6)	36.8 (32.7, 41.4)	40.7 (36.6, 45.4)	40.8 (37.1, 45.0)	0.0913

Stillbirths, (rate/1000 births)	29.3 (25.6, 33.5)	21.5 (18.6, 24.9)	21.8 (19.1, 25.0)	21.1 (18.6, 23.8)	0.0024

NMR < 7 days, (rate/1000 live births)	18.7 (15.4, 22.6)	15.7 (12.4, 19.8)	19.4 (15.3, 24.6)	20.4 (16.9, 24.6)	0.0280

Neonatal mortality < 28 days, (rate/1000 live births)	24.5 (20.7, 29.0)	19.0 (15.6, 23.2)	24.0 (19.3, 29.9)	24.4 (20.8, 28.5)	0.3139

In Belgaum, the highest proportion of neonatal deaths was attributed to birth asphyxia (with a range of 31.8% to 38.7%), followed by low birth weight/prematurity, and other causes (Table 5). In Nagpur, low birth weight/prematurity was recorded as the leading cause of neonatal death (with a range of 34.8% to 44.4%), followed by birth asphyxia, and other causes. In contrast, at the Nagpur site, NMR associated with birth asphyxia and low birth weight/prematurity increased over this period from 20% and 34.8% to 28.8% and 44.2% respectively.

**Table 5:** Cause of neonatal mortality by site

	Belgaum				Nagpur			
	
	2010	2011	2012	2013	2010	2011	2012	2013
Cause of Neonatal Deaths < 28 Days, N (%)	390	377	367	333	230	169	214	226

Birth Asphyxia	151 (38.7)	138 (36.6)	130 (35.4)	106 (31.8)	46 (20.0)	41 (24.3)	43 (20.1)	65 (28.8)

Low Birth Weight/Prematurity	113 (29.0)	107 (28.4)	108 (29.4)	105 (31.5)	80 (34.8)	75 (44.4)	82 (38.3)	100 (44.2)

Infection	31 (7.9)	33 (8.8)	27 (7.4)	30 (9.0)	30 (13.0)	15 (8.9)	21 (9.8)	12 (5.3)

Malformation	8 (2.1)	12 (3.2)	17 (4.6)	16 (4.8)	9 (3.9)	6 (3.6)	10 (4.7)	7 (3.1)

Other	73 (18.7)	74 (19.6)	79 (21.5)	67 (20.1)	31 (13.5)	15 (8.9)	39 (18.2)	37 (16.4)

Don't Know	14 (3.6)	10 (2.7)	6 (1.6)	8 (2.4)	34 (14.8)	17 (10.1)	18 (8.4)	5 (2.2)

## Discussion

**Figure 3:**
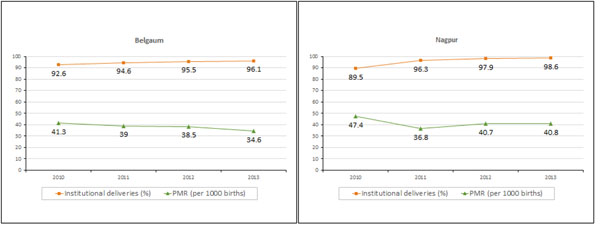
Trends of Institutional Delivery (%) and Perinatal Mortality (per 1000 births)

The MNHR represents a large prospective population-based study conducted in two districts in India. The results from Belgaum and Nagpur indicate a significant increase in institutional delivery that was accompanied by a substantial decline in perinatal mortality between 2010 and 2013 (Figure 3). Substantial declines in stillbirths in both Belgaum and Nagpur were also observed over this period. In Belgaum, the large reduction in perinatal mortality is primarily attributed to the decline in the stillbirth rate, as the early neonatal mortality rate declined only marginally during this period. Some of the reduction in stillbirths and perinatal mortality may be associated with socio-demographic changes that were observed over time, including the shift in age and parity, higher education and fewer deliveries that occurred over time. We posit that some of the decline in stillbirth and perinatal mortality, however, may be attributed to more direct causes, including the resuscitation training of birth attendants, both as part of the Global Network's Emergency Obstetric and Neonatal care (EmONC), trial and the American Academy of Pediatrics Helping Babies Breathe (HBB) studies, and the Ministry of Health's initiative of Navajat Shishu Suraksha Karyakram (NSSK) national basic newborn care and resuscitation program [20-23]. With stimulation and resuscitation, we posit that these programs improved the survival of newborns with simple birth asphyxia, and thus fewer newborns were classified as stillbirths. Birth asphyxia remains a leading cause of death in both sites, and indicates further efforts to improve obstetric care and to provide first minute bag and mask resuscitation are needed. Yet, very early and early neonatal mortality rates rose in Nagpur, and thus there was a smaller decline in perinatal mortality in Nagpur than in Belgaum. Still, unlike the quasi-experimental comparisons of India’s Janani Suraksha Yojana conditional cash transfer program [13], virtually no improvement was observed in early or 28-day neonatal survival in either location. The decline in stillbirth but not neonatal mortality rates may be mediated by the increase in cesarean sections, responsive to fetal distress, and is consistent with observations from Bangladesh [15]. In Bangladesh, the demand side financing program was associated with an increase in cesarean section rates, but otherwise with little or no improvement in quality of care. Low birth weight remains a leading cause of neonatal death, and the lack of effect on neonatal mortality is consistent with persistent high mortality rates observed for very low birth weight infants [24] and the need to improve quality of care for newborns.

The trends of lower stillbirth rates associated with increasing cesarean rates in lower and middle income countries is also consistent with observations from multi-country World Health Organization data [25,26]. Improving obstetric care is critical to prevention of birth asphyxia [27,28-30]. The EmONC research trial that both the Belgaum and Nagpur sites as well as the other Global Network sites participated in also found limited influence on institutional quality of care and concluded that improving pregnancy outcomes requires more infrastructural preparedness than provider training and community mobilization alone can provide [27]. The observed reduction in stillbirth rates may also be associated with more babies receiving resuscitation. The decline in asphyxia related mortality in Belgaum supports this speculation. Even though there was a decline in bag and mask resuscitation in Belgaum, resuscitative stimulation, which is not assessed in the registry, likely increased in the area's HBB projects [19]. While the increase in cesarean section rates suggests that more women are being identified with pregnancy complications through institutional delivery, the increase in community NMR indicates the continued delivery of high risk or complicated cases in home births. Caste disparities continue to exist, with women of lower castes continuing to have higher rates of home birth [31].

The increase in health care facility deliveries is consistent with the patterns observed in all 20 Belgaum clusters of which only the 16 providing 48 months of data were included in the analyses presented. The increase in institutional deliveries is also similar to that observed in other countries using various schemes to increase institutional delivery. Unlike the special community and facility-based surveys to assess their program effects [32,33], the MNHR provides standardized high-quality data from a prospective, surveillance system that captures over 99% of permanent resident pregnancies and their outcomes [19].

## Conclusions

The MNHR data from Belgaum and Nagpur, in Southern and Central India, indicate a significant increase in institutional, and particularly hospital, deliveries between 2010 and 2013. This was accompanied by substantial reductions in perinatal mortality, stillbirth, and, in Belgaum, a declining trend in very early and early neonatal mortality. We posit that some of this decline was associated with increasing caesarean section rates and neonatal resuscitation. With increased emphasis on stimulation and resuscitation, it is likely that resuscitation training programs, besides improving survival of newborn babies with birth asphyxia, may also have improved recognition that some non-breathing infants are not stillbirths. Thus, fewer newborns may have been classified as stillbirths. Birth asphyxia remains a leading cause of death in both sites, and indicates further efforts to provide quality obstetric care and first minute bag and mask resuscitation are needed. However, the lack of improvement in 28 day neonatal mortality indicates that further efforts are needed to improve quality of care and, perhaps, to ensure that all women experiencing complications seek and receive timely appropriate skilled birth attendance and institutional delivery to further advance perinatal and neonatal survival.

## List of abbreviations used

EmONC: Emergency Obstetric and Neonatal Care; HBB: Helping Babies Breathe; MDG: Millennium Development Goal; MNH: Maternal Newborn Health Registry; MOH: Ministry of Health; NMR: Neonatal mortality rate; NSSK: Navajat Shishu Suraksha Karyakram; PMR: Perinatal mortality rate.

## Competing interests

The authors declare no competing interests.

## Author’s contributions

SSG conceived the idea for this study. SSG, NG and NLS wrote the first draft. JLM and DDW performed data analyses. SSG, NG, MSS, SSV, AAM, AP, NLS,PLH, MKT, EMM and RLG read and approved the final manuscript.

## Peer review

Reviewer reports for this article can be found in Additional file 1.

## Supplementary Material

Additional file 1 Click here for file

## Declarations

This article has been published as part of *Reproductive Health* Volume 12 Supplement 2, 2015: Research reports from the NICHD Global Network for Women’s and Children’s Health Research Maternal and Newborn Health Registry. The full contents of the supplement are available online at http://www.reproductive-health-journal.com/supplements/12/S2. Publication of this supplement was supported by grants from the Eunice Kennedy Shriver National Institute of Child Health and Human Development to RTI International.

## References

[B1] Liu L Oza S Hogan D Perin J Rudan I Lawn EJ Global, regional, and national causes of child mortality in 2000–13, with projections to inform post-2015 priorities: an updated systematic analysis Lancet 2014 6736 9966 430 440 25280870 10.1016/S0140-6736(14)61698-6

[B2] Lawn JE Blencowe H Pattinson R Cousens S Kumar R Ibiebele I Stillbirths: Where? When? Why? How to make the data count? Lancet 2011 377 9775 1448 1463 10.1016/S0140-6736(10)62187-3 21496911

[B3] Mason E McDougall L Lawn JE Gupta A Claeson M Pillay Y From evidence to action to deliver a healthy start for the next generation Lancet 2014 384 9941 455 467 10.1016/S0140-6736(14)60750-9 24853599

[B4] Lawn JE Cousens S Zupan J Lancet Neonatal Survival Steering Team 4 million neonatal deaths: when? Where? Why? Lancet 2005 365 9462 891 900 10.1016/S0140-6736(05)71048-5 15752534

[B5] Ronsmans C Graham WJ Lancet Maternal Survival Series steering group Maternal mortality: who, when, where, and why Lancet 2006 368 9542 1189 200 10.1016/S0140-6736(06)69380-X 17011946

[B6] Li XF Fortney JA Kotelchuck M Glover LH The postpartum period: the key to maternal mortality Int J Gynaecol Obstet 1996 54 1 1 10 10.1016/0020-7292(96)02667-7 8842811

[B7] United Nations General Assembly United Nations Millennium Declaration 2000 A/RES/55/2 edn New York, NY Available at: http://www.un.org/millennium/declaration/ares552e.htm. Accessed August 25, 2014

[B8] WHO. Making pregnancy safer: the critical role of the skilled attendant A joint statement by WHO, ICM and FIGO 2004 Geneva, Switz WHO 18

[B9] Campbell OMR Graham WJ Strategies for reducing maternal mortality: getting on with what works Lancet 2006 368 9543 1284 1299 10.1016/S0140-6736(06)69381-1 17027735

[B10] Gupta SK Pal DK Tiwari R Garg R Shrivastava AK Sarawagi R Impact of Janani Suraksha Yojana on institutional delivery rate and maternal morbidity and mortality: an observational study in India J Health Popul Nutr 2012 30 4 464 471 23304913 10.3329/jhpn.v30i4.13416 PMC3763618

[B11] Maternal Health Division, Ministry of Health and Family Welfare, Government of India Guidelines for Skilled Attendance at Birth by Auxiliary Nurse Midwives, Lady Health Visitors & Staff Nurses 2010 http://nrhm.gov.in/images/pdf/programmes/maternal-health/guidelines/sba_guidelines_for_skilled_attendance_at_birth.pdf Accessed January 9, 2015

[B12] Ministry of Health and Family Welfare, Government of India Navjaat Shishu Suraksha Karyakram: Basic Newborn Care and Resuscitation Program 2009

[B13] Lim SS Dandona L Hoisington JA James SL Hogan MC Gakidou E India's Janani Suraksha Yojana, a conditional cash transfer programme to increase births in health facilities: an impact evaluation Lancet 2010 375 9730 2009 2023 10.1016/S0140-6736(10)60744-1 20569841

[B14] Lagarde M Haines A Palmer N The impact of conditional cash transfers on health outcomes and use of health services in low and middle income countries Cochrane Database of Systematic Reviews 2009 4 CD008137 19821444 10.1002/14651858.CD008137 PMC7177213

[B15] Hatt L Nguyen H Sloan N Miner S Magvanjav O Sharma A Chowdhury J Islam M Wang H Economic Evaluation of Demand-Side Financing (DSF) for Maternal Health in Bangladesh [Draft] Review, Analysis and Assessment of Issues Related to Health Care Financing and Health Economics in Bangladesh, Abt Associates Inc 2010 Bethesda, MD

[B16] http://www.census2011.co.in/census/district/244-belgaum.html Accessed March 27, 2015

[B17] http://www.census2011.co.in/census/district/343-nagpur.html. Accessed March 27, 2015

[B18] International Institute for Population Sciences (IIPS) and Macro International National Family Health Survey (NFHS-3) 2007 I India: Mumbai: IIPS 2005 06 Chapter 3, pp. 53-76

[B19] Goudar SS Carlo WA McClure EM The Maternal and Newborn Health Registry Study of the Global Network for Women's and Children's Health Research Int J Gynaecol Obstet 2012 118 3 190 193 10.1016/j.ijgo.2012.04.022 22738806 PMC3417109

[B20] 2015 http://www.nihfw.org/pdf/NCHRC-Publications/NavjaatShishuTrgMan.pdf Accessed January 9

[B21] Goudar SS Dhaded SM McClure EM Derman RJ Wright LL Bellad RM Kodkany BS Moore JM Carlo WA ENC training reduces perinatal mortality in Karnataka India J Matern Fetal Neonatal Med 2012 25 6 568 574 10.3109/14767058.2011.584088 PMC13280768 21793707

[B22] Goudar SS Somannavar MS Clark R Lockyer JM Revankar AP Fidler HM Stillbirth and Newborn Mortality in India After Helping Babies Breathe Training Pediatrics 2013 131 2 e344 e352 10.1542/peds.2012-2112 23339215

[B23] Carlo WA Goudar SS Parida S Jehan I Tshefu A Chomba E Newborn care training and perinatal mortality in communities N Engl J Med 2010 362 7 614 623 10.1056/NEJMsa0806033 20164485 PMC3565382

[B24] Carlo WA Goudar SS Jehan I Chomba E Tshefu A Garces A High mortality rates for very low birth weight infants in developing countries despite training Pediatrics 2010 126 5 e1072 e1080 10.1542/peds.2010-1183 20937655 PMC3918943

[B25] Goldenberg RL1 McClure EM Bann CM The relationship of intrapartum and antepartum stillbirth rates to measures of obstetric care in developed and developing countries Acta Obstet Gynecol Scand 2007 86 11 1303 1109 10.1080/00016340701644876 17963057

[B26] McClure EM Goldenberg RL Bann CM Maternal mortality, stillbirth and measures of obstetric care in developing and developed countries Int J Gynaecol Obstet 2007 96 2 139 146 10.1016/j.ijgo.2006.10.010 17274999

[B27] Pasha O McClure EM Wright LL Saleem S Goudar SS Chomba E A combined community- and facility-based approach to improve pregnancy outcomes in low-resource settings: a Global Network cluster randomized trial BMC Med 2013 11 215 10.1186/1741-7015-11-215 24090370 PMC3853358

[B28] Goldenberg RL McClure EM Jobe AH Kamath-Rayne BD Gravette MG Rubens CE Commentary: Stillbirths and neonatal mortality as outcomes Int J Gynecol Obstet 2013 122 3 230 233 http://dx.doi.org/10.1016/j.ijgo.2013.06.020 10.1016/j.ijgo.2013.04.008 PMC4349406 24050480

[B29] Kamath-Rayne BD Griffin JB Moran K Jones B Downs A McClure EM Resuscitation and obstetrical care to reduce intrapartum-related neonatal death: A MANDATE study Matern Child Health J 2015 10.1007/s10995-015-1699-9 25656720

[B30] Bhutta ZA Yakoob MY Lawn JE Rizvi A Friberg I Weissman E Stillbirths: What works? How much difference can we make and at what cost? Lancet 2011 377 9776 1523 1538 10.1016/S0140-6736(10)62269-6 21496906

[B31] Adamson PC Krupp K Niranjankumar B Freeman AH Khan M Madhivanan P Are marginalized women being left behind? A population-based study of institutional deliveries in Karnataka, India BMC Public Health 2012 12 30 10.1186/1471-2458-12-30 22240002 PMC3269389

[B32] Ir P Horemans D Souk N van Damme W Using targeted vouchers and health equity funds to improve access to skilled birth attendants for poor women: a case study in three rural health districts in Cambodia BMC Pregnancy Childbirth 2010 10 1 10.1186/1471-2393-10-1 20059767 PMC2820432

[B33] Witter S Khadka S Nath H Tiwari S The national free delivery policy in Nepal: early evidence of its effects on health facilities Health Policy and Planning 2011 26 ii84 ii91 22027923 10.1093/heapol/czr066

