# Supplementary material for: Institutional deliveries and perinatal and neonatal mortality in Southern and Central India
Source: Reprod Health. 2015 Jun 8;12(Suppl 2):S13. doi: 10.1186/1742-4755-12-S2-S13 (PMC4464025; doi:10.1186/1742-4755-12-S2-S13)
Supplement: Additional file 1 [file 1742-4755-12-S2-S13-S1.pdf]

Referee's comments to the authors– this sheet WILL be seen by the author(s) and published with the article

Title	Institutional Deliveries and Perinatal and Neonatal Mortality in Southern and Central India
Author(s)	Shivaprasad S Goudar, Norman Goco, Manjunath S Somannavar, Sunil S Vernekar, Ashalata A Mallapur, Janet L Moore, Dennis D Wallace, Nancy L Sloan, Archana Patel, Patricia L Hibberd, Marion Koso-Thomas, Elizabeth M McClure, and Robert L Goldenberg
Referee's name	William Keenan

When assessing the work, please consider the following points, where applicable:

1. Is the question posed by the authors new and well defined?
2. Are the methods appropriate and well described, and are sufficient details provided to replicate the work?
3. Are the data sound and well controlled?
4. Does the manuscript adhere to the relevant standards for reporting and data deposition?
5. Are the discussion and conclusions well balanced and adequately supported by the data?
6. Do the title and abstract accurately convey what has been found?
7. Is the writing acceptable?

Please make your report as constructive and detailed as possible in your comments so that authors have the opportunity to overcome any serious deficiencies that you find and please also divide your comments into the following categories:

- Major Compulsory Revisions (which the author must respond to before a decision on publication can be reached)
- Minor Essential Revisions (such as missing labels on figures, or the wrong use of a term, which the author can be trusted to correct)
- Discretionary Revisions (which are recommendations for improvement but which the author can choose to ignore)

Where possible please supply references to substantiate your comments.

When referring to the manuscript please provide specific page and paragraph citations where appropriate.

General comments:

Major compulsory revisions:

Minor essential revisions:
Page 4, Background, line 4: "amny" is probably more accurate than "most".

Page 7, parra 2, line 2: Place period following "Nagpur". New sentence beginning with "The".

Page 8, para 3 and 4: Figures and Tables in English usage are passive. They cannot present or illustrate. Change use to passive such as "The PMR and ….are illustrated in figures 1 and 2."

Page 10, Discussion, para 1, line 7: "Some of this reduction (many data points being discussed—

(continue on the next sheet)

Continued: should add an identifier “stillbirth rate”
Same paragraph—add some discussion about change in classification form a still birth to a live birth. This is the first time it comes up in the manuscript and the issue should be clarified for the reader here.

Page 11, Conclusions, live 1: These are household data plus the delivery location data not just household data forming this conclusion. Reword.

Table 1, left hand column: label “Maternal age, N (%) but only N is shown. Change label Same for items of “Maternal Education” and “At least one ANC visit” Other rows seem to show % but left column is not properly labeled

TABLE 2, left hand column: Label says N(%) three places but only N are shown in table. Some other rows give a % but labeling not complete in left column.

Tables 4 and 5: similar issues.

Referee's comments to the authors– this sheet WILL be seen by the author(s) and published with the article

Title	Institutional Deliveries and Perinatal and Neonatal Mortality in Southern and Central India
Author(s)	Shivaprasad S Goudar, Norman Goco, Manjunath S Somannavar, Sunil S Vernekar, Ashalata A Mallapur, Janet L Moore, Dennis D Wallace, Nancy L Sloan, Archana Patel, Patricia L Hibberd, Marion Koso-Thomas, Elizabeth M McClure, and Robert L Goldenberg
Referee's name	Steve Entman

When assessing the work, please consider the following points, where applicable:

1. Is the question posed by the authors new and well defined?
2. Are the methods appropriate and well described, and are sufficient details provided to replicate the work?
3. Are the data sound and well controlled?
4. Does the manuscript adhere to the relevant standards for reporting and data deposition?
5. Are the discussion and conclusions well balanced and adequately supported by the data?
6. Do the title and abstract accurately convey what has been found?
7. Is the writing acceptable?

Please make your report as constructive and detailed as possible in your comments so that authors have the opportunity to overcome any serious deficiencies that you find and please also divide your comments into the following categories:

- Major Compulsory Revisions (which the author must respond to before a decision on publication can be reached)
- Minor Essential Revisions (such as missing labels on figures, or the wrong use of a term, which the author can be trusted to correct)
- Discretionary Revisions (which are recommendations for improvement but which the author can choose to ignore)

Where possible please supply references to substantiate your comments.

When referring to the manuscript please provide specific page and paragraph citations where appropriate.

General comments: The fundamental premise behind the study is valid and necessary: a two-pronged attack on maternal and perinatal/neonatal mortality death has been implemented through public health efforts in India. The authors state that, "to date, few studies have assessed the potential impact of these activities on maternal, perinatal and (*sic)* maternal mortality." They cite none and regrettably, their prospective, self-defined "descriptive" study, despite lots of data, provides only minimal information. In the end, they have told us about patterns of migration to hospitals and clinics for birthing and shifts in birth attendants. There are a lot of numbers about time frame in which perinates/neonates die. It is hard to track the data to verify (more later). There is very little discussion about what this all means for the health system, what has succeeded, what has failed, what more should be done. As best I can tell, their unstated bottom line is that increased numbers of cesarean sections rescued distressed fetuses from stillbirth and perhaps even early neonatal death, but in the end, survival past 28 days was unchanged.

Major compulsory revisions:

1. **General issues about the populations**
    a. **We need some info about the characteristics of the two different population groups,**

(continue on the next sheet)

Continued:

what is different about the States? why not aggregate them as one cohort?

b. You used all consenting women? How many didn't consent? Did that bias the results?

c. There were 8/24 study clusters in Belgaum not included because of incomplete data. Was there any systematic bias that drove that variance?

2. You compared trend over time, focusing on the obstetrical parameters. You mentioned some covariables, but ignored others that I suspect were both clinically and statistically significant. They certainly warrant mention and discussion. Specifically, not only did the frequency of low education decline over time but the frequency of higher education increased greatly over time. This was consistent with the shift in age and parity. There were 2000 fewer births; 1600 of the decrement was among uneducated women and 1400 among primary educated, offset by increases 900 among high school and university women. The first step in improving pregnancy outcomes is to improve the socioeconomic status of the population. That happened somewhat independently of the public health initiative being studied. Notably, in Belgaum, where there was the biggest demographic change, there was the largest decline in number of deliveries.

3. As stated in my general comments, I had some trouble following the numbers. I am not a biostatistician and I understand that I can't do tests for "trends across time with an orthogonal polynomial linear contrast" model in my head. On the other hand, I used as much raw data that you provided to try to track the rates presented and I can't reproduce the rates reported in Table 4.

For example, I would have thought that since both stillbirth rate and early neonatal mortality rates are normalized to a denominator of 1000, adding the numerators should equal the Perinatal Mortality Rate for that site and year. Yet for Nagpur in 2013, 21.1+20.4=41.5, not the reported 40.8. This is within confidence limits but having raw numbers to match up would help make sense of the data.

Having raw data helped review the later discussion about Belgaum neonatal death causes and bag and mask resuscitation. In fact, the number and rate of resuscitation events declined significantly in 2013 and while asphyxia was the leading cause of death, it has trended lower as infection and unknown have increased. Raw numbers as well as rates are helpful. But examination of the data by the authors and comment about what it does mean and what it could mean for future program development would be far more helpful.

Minor issue:

Last sentence on bottom of page 5 mentions women "who have undergone sterilization and are likely to conceive". This is rather confusing at best.
